# Improved Neuromuscular Performance in Low-Load vs. Moderate-Load Resistance Training Among Young Elite Swimmers

**DOI:** 10.3390/sports14060247

**Published:** 2026-06-17

**Authors:** David Rodríguez-Rosell, Henrique Pereira Neiva, Daniel Almeida Marinho, Juan Manuel Yáñez-García, Andrés Rojas-Jaramillo, Juan José González-Badillo, Mário Cardoso Marques

**Affiliations:** 1 Physical Performance & Sports Research Center, Universidad Pablo de Olavide, 41013 Seville, Spain; davidrodriguezrosell@gmail.com; 2 Department of Sport and Informatics, Universidad Pablo de Olavide, 41013 Seville, Spain; jjgbadi@gmail.com; 3 Department of Sport Sciences, University of Beira Interior, 6201-001 Covilhã, Portugal; hpn@ubi.pt (H.P.N.); dmarinho@ubi.pt (D.A.M.); mariomarques@mariomarques.com (M.C.M.); 4 Research Center in Sports Sciences, Health Sciences and Human Development—CIDESD, 6201-001 Covilhã, Portugal; 5 Department of Communication and Education, Universidad Loyola Andalucía, 41704 Seville, Spain; 6 Educational and Pedagogical Studies and Research Group (GEIEP), Corporación Universitaria Minuto de Dios, Medellín 050034, Colombia; andres.rojasj@udea.edu.co

**Keywords:** land-based strength training, swimming performance, full-squat, jump, bench press

## Abstract

Resistance training (RT) is commonly used to enhance neuromuscular performance and sprint swimming outcomes. However, the optimal relative load for elite junior swimmers remains unclear. In particular, little is known about whether very low relative loads can elicit meaningful adaptations while minimizing neuromuscular fatigue in athletes exposed to high concurrent training demands. Therefore, the aim of this study was to compare the effects of two land-based RT programs differing only in relative load intensity (40–50% vs. 55–65% 1RM), performed with maximal intended concentric velocity, on strength, jumping ability, and 50 m freestyle swimming performance in elite junior swimmers. Eighteen elite junior swimmers (15.6 ± 0.9 years) from a national high-performance program were randomly assigned to a low-load (40–50% 1RM; *n* = 9) or moderate-load (55–65% 1RM; *n* = 9) group. Both groups completed an 8-week RT program (2 sessions·week^−1^) with identical exercise selection, volume, execution velocity, and in-water training load. Neuromuscular performance (countermovement jump, squat, bench press, and pull-up strength) and swimming performance (50 m freestyle from the starting block and in-water start) were assessed pre- and post-intervention. Both RT protocols improved squat and bench press strength and 50 m freestyle performance, whereas significant improvements in countermovement jump, pull-up strength, and maximal pull-up repetitions were observed only in the low-load group. Significant group × time interactions were found for countermovement jump, maximal number of pull-up repetitions, and 50 m freestyle performance from the starting block, indicating more favorable changes over time in the low-load group. In conclusion, both low- and moderate-load high-velocity RT improved neuromuscular and 50 m freestyle performance outcomes in elite junior swimmers. However, the low-load RT (40–50% 1RM) appeared to provide additional benefits in specific outcomes (i.e., jumping, pull-ups, and 50 m performance from the starting block). These findings suggest that relatively low loads may be a practical alternative to moderate-load RT in high-volume swimming training environments.

## 1. Introduction

Competitive swimming performance depends on the swimmer’s ability to generate high levels of propulsion while minimizing hydrodynamic resistance across race distances ranging from short sprints to long endurance events [1,2]. In sprint swimming events such as the 50 m front crawl, performance is largely determined by the capacity to produce force rapidly during key race segments, including the start, turn, and free-swimming phases [3,4]. Accordingly, neuromuscular characteristics, particularly the rate of force development of the upper and lower limbs, have been identified as important determinants of short-distance swimming performance [5,6].

Several studies have reported significant associations between dry-land strength measures and sprint swimming performance, supporting the inclusion of resistance training (RT) as a complementary strategy to in-water training in competitive swimmers [7,8]. Lower-limb strength has been strongly related to start and turn performance, as these phases require rapid force production and high mechanical power output [9,10]. Similarly, upper-limb strength plays a crucial role in propulsion during the stroke phase, with muscles such as the latissimus dorsi and pectoralis major contributing substantially to force generation in freestyle swimming [11,12].

As a consequence, the integration of dry-land RT into swimming programs has become common practice across competitive levels [13,14]. When appropriately designed, RT could enhance neuromuscular performance, improve sport-specific actions, and potentially reduce injury risk in youth and elite athletes [5,15]. The effectiveness of RT depends on exercise selection and key programming variables such as relative load, training volume, voluntary movement velocity, and fatigue management [16].

Traditionally, RT programs for swimmers have emphasized moderate- to high-load training (≥70% 1RM) to increase maximal strength and muscle mass [11,17]. Although such approaches may improve maximal force production (i.e., 1RM), these types of loads are frequently associated with greater neuromuscular fatigue and prolonged recovery periods, which may compromise the quality and consistency of in-water training sessions [18], particularly in young swimmers exposed to high weekly in-water training volumes [19]. This issue is especially relevant in elite junior swimmers, for whom technical development and high-frequency swimming practice are critical components of long-term performance progression [20,21].

RT adaptations are strongly influenced by the relative load applied during exercise execution, as different intensity zones elicit distinct mechanical, neural, and metabolic stimuli even when exercises and volumes are matched [22]. While higher relative loads have traditionally been associated with maximal strength and hypertrophic adaptations, accumulating evidence indicates that meaningful improvements in strength could be achieved across a wide spectrum of relative loads, provided that training is performed with high intent and appropriate volume control [22,23]. In particular, low- to moderate-load RT (≈30–60% 1RM) executed at maximal concentric velocities may contribute to neuromuscular adaptations relevant to rapid force production, including improvements in RFD and motor unit recruitment efficiency [24].

From a mechanical and neural perspective, training with lower relative loads allows athletes to achieve higher movement velocities, which may enhance the specificity of neuromuscular adaptations to high-speed sporting actions [25,26]. In addition, lower-load protocols are typically associated with lower acute mechanical and neuromuscular demands, which may facilitate recovery and help maintain training quality during subsequent sessions [18]. This characteristic is particularly relevant in swimming, where athletes are exposed to high weekly training volumes and limited recovery time between sessions [13,21].

In recent years, increasing attention has been given to the concept of applying the minimum effective dose of RT, emphasizing the identification of the lowest combination of training loads and volume capable of eliciting positive adaptations while minimizing unnecessary fatigue [27]. This approach is especially important in young athletes, for whom excessive training loads may increase injury risk, interfere with technical skill acquisition, and compromise long-term athletic development [15,28]. Accordingly, RT strategies based on relatively low intensities, such as ~40% 1RM, may represent a safe and efficient option for enhancing neuromuscular performance while maintaining compatibility with high-volume sport-specific training [29].

Although several studies conducted in team sports have observed that low-load, low-volume RT produces improvements in strength and sport-specific performance comparable to those achieved with higher relative loads [30], the applicability of these findings to competitive swimming remains unclear. Importantly, no longitudinal studies to date have systematically examined the effects of different ranges of relative load intensity during RT in elite swimmers, despite the relevance of this variable for modulating movement velocity, fatigue, and training efficiency [16]. Therefore, the novelty of the present study lies in examining whether a low-load, high-velocity RT program (40–50% 1RM) can induce meaningful neuromuscular and sprint swimming adaptations in elite junior swimmers, while isolating relative load as the only manipulated training variable. This has practical relevance because coaches working with competitive swimmers must often develop strength and high-velocity neuromuscular qualities without adding excessive training demands to already high-volume swimming programs.

Therefore, the purpose of the present study was to analyze the effects of two RT programs differing only in relative load (40–50% vs. 55–65% 1RM), performed with maximal intended concentric velocity, on strength, jumping ability, and sprint swimming performance in elite junior swimmers. All other training variables, including exercise selection, volume, execution velocity, and in-water training load, were strictly controlled and matched. We hypothesized that both loading conditions would induce meaningful improvements in strength, jumping ability, and 50 m freestyle performance in elite junior swimmers. However, given the higher movement velocities allowed by lower relative loads, we further hypothesized that the low-load, high-velocity condition (40–50% 1RM) would elicit more favorable changes in selected high-velocity neuromuscular and sprint swimming outcomes.

## 2. Materials and Methods

### 2.1. Participants

A group of 20 elite junior swimmers (8 females, 12 males) competing in the Portuguese Swimming Championships, European Junior Swimming Championships, and World Junior Championships volunteered to participate in this study. During the training intervention, 2 women participants were excluded from the study because they were injured or were absent from the post-testing session. As a result, the training program was completed by 18 swimmers (6 female and 12 male). Participant characteristics are displayed in Table 1. An a priori power analysis was conducted using G*Power software (version 3.1.9.7 Heinrich-Heine-Universität Düsseldorf, Düsseldorf, Germany) for an F test repeated-measures ANOVA, within–between interaction. The following parameters were used: effect size f = 0.25, α = 0.05, power = 0.85, two groups, two measurements, an assumed correlation among repeated measures of 0.80, and nonsphericity correction ε = 1. Under these assumptions, the required total sample size was 18 participants, with an actual power of 0.88. Nevertheless, because the sample size is sensitive to the assumed correlation among repeated measures, the relatively small final sample should be considered when interpreting the between-group findings.

Participants had a minimum of 5 years of swimming training experience and were injury-free for at least 3 months before engaging in this research. In addition, both groups had at least 3 months of previous strength training experience. Although no formal dietary records were collected before training or testing sessions, all participants remained in the same high-performance center throughout the intervention, where training, feeding, and rest conditions were kept identical for all swimmers. Before the experiment, all swimmers underwent physical examination by a physician and were cleared of any medical disorders that might limit their participation in the investigation. Afterward, the participants were matched according to the results of their 50 m swimming test from the starting block, and then randomly assigned into either the low-load (G40–50%) or the moderate-load (G55–65%) group (*n* = 3 females and 6 males each). None of the swimmers were taking exogenous anabolic−androgenic steroids, drugs, or other supplements that may affect physical performance or hormonal balance during the study. This was controlled by the medical department and by one of the researchers. The coaches and parents were informed about the different test procedures and training sessions to be performed during the study, and parental/guardian consent was obtained for all participants under the age of 18. All subjects were informed about the experimental procedures and potential risks before providing written informed consent. This study was approved by the Institutional Review Committee Board of the local Committee for Medical Research Ethics and current Portuguese law and regulations (Approval code: EH-1/2015, Approval date: 28 January 2015), and it was carried out according to the Declaration of Helsinki.

### 2.2. Study Design

This was an experimental longitudinal study that analyzed the effects of low-load versus moderate-load RT programs (40–50% vs. 55–65% 1RM) on jump, strength, and swimming performance. Both groups performed 6–8 swimming practice sessions per week, combined with an RT regimen including upper- and lower-body exercises 2 times a week (72 h rest) during an 8-week training period. The characteristics of the RT program for both groups were identical, aside from the differences in the relative load used during the full squat and bench press exercise. All swimmers were assessed before (pre-training) and after (post-training) the training period using the following tests: countermovement jump (CMJ) test; progressive loading test in the full squat (SQ), bench press (BP), and pull-up (PU) exercises; maximal number of repetitions (MNR) in the PU exercise; and 50 m swim time from the starting block (T50_SB) and from inside the pool (T50_IP). Prior to the study, the swimmers completed a pre-season training routine for four consecutive weeks to familiarize themselves with all the tests and training exercises. All athletes were in good overall condition and were adequately familiarized with all the procedures and exercise techniques. All testing and training sessions were conducted in a laboratory under the direct supervision of the investigators and under controlled environmental conditions (~21 °C and ~60% humidity).

### 2.3. Testing Procedures

An introductory session was used to collect anthropometric assessments, conduct medical examinations, and familiarize participants with the testing protocols, during which they were well-rested and in a fasted state. After medical screening and body composition measurements, the participants performed an exercise session with light loads (~30% 1RM) and a low-volume (2–3 sets of 4–8 repetitions per set), during which the researchers emphasized proper technique to ensure their familiarity with the protocol for each testing session. The CMJ and strength tests (progressive loading test in the SQ, BP and PU exercises) and 50 m swim tests were assessed in 2 sessions with 48 h of rest in between. The testing sessions were conducted in the afternoon (16:00–18:00 h) under similar environmental conditions (~22–24 °C and ~55–65% humidity) for all participants. At least 1 day before the swim tests, the participants were prohibited from performing fatiguing training sessions. Strong verbal encouragement was provided during all tests to motivate the participants to exert maximal effort.

#### 2.3.1. Anthropometric Assessment

The participants were dressed in a skin-tight swimsuit and a swimming cap during all measurements. Height and body mass were measured using a medical stadiometer and scale, respectively (Seca 710, Ltd., Hamburg, Germany). Wingspan was measured as the length between the tips of both middle fingers, with both arms abducted horizontally and perpendicular to the body’s upright position. The skinfold thickness at eight sites (i.e., subscapular, triceps, bicep, iliac crest, supraspinal, abdominal, front thigh, and medial calf) was measured using standardized Harpenden skinfold calipers. Each measurement was taken twice unless the readings differed by ≥10%, in which case a third measurement was taken. The skinfolds were averaged to represent the skinfold thickness. Measurements were taken by one of two trained researchers, and the same researcher was used for all skinfold measurements on a single participant.

#### 2.3.2. Vertical Jump Test

A countermovement jump test (CMJ) was performed with the participants standing in an upright position on an infrared timing system (Optojump System, Microgate, Bolzano, Italy), with hands placed on the hips to avoid arm swings. In a single sequence, participants were asked to perform a fast downward movement immediately, followed by a fast upward vertical movement as high as possible, and then instructed to land in an upright position with bent knees. The vertical jump height (h) of the center of gravity of the body was calculated using flight time (t) and acceleration due to gravity (g) as follows: h = t^2^ × g/8. To ensure that the jump was completely vertical, the subjects were required to take off and land within the same area; otherwise, the jump was not considered. After a warm-up consisting of 2 sets of 10 squats without external load and 5 CMJs (with 20 s rest intervals), each participant performed 3 maximal CMJs with their hands on their hips, separated by 1 min rest intervals. The averages of the highest and lowest values were retained for analysis. The coefficient of variation (CV) for test−retest reliability was 1.7%, and the intraclass correlation coefficient (ICC) was 0.996 (95% CI: 0.992–0.999).

#### 2.3.3. Progressive Loading Test for the Bench Press (BP) and Full Squat (SQ)

These tests had the following objectives: (1) to estimate the strength of the lower and upper limbs, and (2) to estimate the weight (kg) that each subject had to use during each training session. Both incremental tests were performed using a Smith machine (Multipower Fitness Line, Peroga, Murcia, Spain). The BP testing protocol was done as previously reported [17]. During the BP, a momentary pause (~1.0 s) at the chest between the eccentric and concentric actions was imposed to minimize the contribution of the rebound effect and allow for more reproducible, consistent measurements. Similarly, the SQ testing protocol was conducted as done in a previous study [31]. Participants started from an upright position, descending in a continuous motion until the posterior thighs and calves made contact, and then, the motion was immediately reversed until the participant ascended back to the starting position. Unlike the eccentric phase, which was performed at a normal, controlled velocity, the concentric phase of both BP and SQ needed to be executed at the maximal intended velocity. The individual positions for the BP (position on the bench and grip width) and SQ (foot position and hand placement on the bar) were measured for each participant so that they could be reproduced in all testing sessions. The initial load was set at 20 kg for all participants for the BP and SQ and then it was gradually increased in 10 kg increments. The tests ended for each participant when the attained concentric mean propulsive velocity (MPV) was lower than 0.6 m·s^−1^ and 0.8 m·s^−1^ for the BP and SQ, respectively, corresponding to ~70% 1RM in both exercises [17,32]. The estimated 1RM was calculated for each individual from the MPV attained against the heaviest load (kg) lifted in the progressive loading tests, as follows: (100 × load)/(−5.961 × MPV2) (50.71 × MPV) + 117 for the SQ [32], and (100 × load)/(8.4326 × MPV2) (73.501 × MPV) + 112.33 for the BP [17]. In addition, the MPV attained against 20, 30, 40, and 50 kg (MPV20, MPV30, MPV40, and MPV50, respectively) were also analyzed.

#### 2.3.4. RM Test and MNR Test in the Pull-Up (PU) Exercise

All PU tests were performed on a standard, stationary, horizontal bar. A complete PU repetition was defined as lifting the body from a full-arm extension hanging position until the chin was above the bar. A self-selected width with a pronated grip (approximately 150% of the biacromial distance) was used throughout the first testing session and recorded, so that it could be repeated in the post-testing session. During each repetition of both tests (1RM and MNR test), the subjects were required to perform the eccentric phase at a controlled velocity, maintain a static position for ∼1 s at the end of this phase, and then begin the concentric phase at the maximal intended velocity upon hearing the command. During the 1RM test, subjects started without additional weight, and the load was gradually increased by 5 kg increments and then by 1 kg additions to precisely determine the 1RM. Regarding the number of repetitions, 3 were executed for light loads, 2 for medium loads, and 1 for heavy loads. The rest intervals between sets ranged from 3 to 5 min. To add extra weight, a specialized belt was used, which could be adjusted around the waist and allowed weights to be attached using a chain. The 1RM was defined as the heaviest load that each subject could properly lift while completing the full range of motion and without any external assistance. Five minutes after completing the progressive loading test, the MNR test was performed. The maximum number of completed repetitions until muscular failure was recorded for each participant. The test was concluded when the participants were unable to raise their chin above the bar or when they paused for more than 2–3 s in the full-arm extension hanging position. The same warm-up protocol, which consisted of 5 min of upper limb joint mobilization exercises and 1 set of 2–5 PU repetitions with no external load, was followed in all testing sessions.

#### 2.3.5. 50 m Swim Tests

The swimmers first warmed up for about 1000 m in water, using the usual swimmer’s strategies [5], including short sprint races. Afterward, the swimmers rested until they felt a complete subjective readiness to perform the maximal effort test (~5 min). The first test started with the swimmers from above the starting block (T50_SB), while the second test started from inside the pool (T50_IP); the start of both tests was signaled using a whistle. The swimmers were instructed to attain the maximal swimming speed as quickly as possible and to maintain it for as long as possible. The race time was recorded using a manual chronometer, starting from the first whistle until the swimmer completed the 50 m test, as signified by touching the wall with one hand. Two 50 m front crawl time trials were performed, with 10 min of recovery in between, for both test modalities. These evaluations were carried out in a 25 m indoor pool with a water temperature of around 27.5 °C [6,8]. Similar to previous studies [8,9], time was recorded by two experienced coaches using a stopwatch (Finis 3x100 Stopwatch, Livermore, CA, USA) and the mean of both measurements was obtained in each trial. The CV and ICC, respectively, were 4.3% and 0.96 (95% CI: 0.93–0.99) for T50_SB, while these were 4.6% and 0.95 (95% CI: 0.92–0.98) for T50_IP.

### 2.4. Training Program

All participants underwent a total of 16 RT sessions over 8 weeks, held twice a week on nonconsecutive days (Monday and Thursday). The descriptive characteristics of the RT are presented in Table 2. The type and order of the exercises, number of sets (3–4 sets), repetitions per set (4–8 repetitions), and recovery time (3 min) were kept identical for both experimental groups in each training session, including the warm-up. The only difference between the groups was the relative load use in the SQ and BP exercises. Thus, the low-load and moderate-load groups trained with relative loads of 40–50% and 55–65% 1RM, respectively. The participants were instructed to perform the concentric phase of each repetition at the maximal intended velocity. To ensure that the exercise prescriptions were properly followed, each training session was supervised by at least two trained researchers. RT included the following exercises: SQ, CMJ, BP, and PU. Exercises were performed in the same order as in Table 2, and all training sets of an exercise had to be completed before proceeding to the next exercise. All RT sessions were performed in the afternoon (16:00–18:00 h), lasting for around 60 min. The loads used by each swimmer in the training exercises were assigned according to the 1RM obtained in the corresponding progressive loading tests during the pre-test. The number of repetitions per set to be performed during each training session for the PU exercise was individually assigned according to the maximal number of repetitions completed in the MNR test. Thus, the number of repetitions per set by each participant progressively increased from 50% to 75%. The participants were instructed to perform all exercises at the maximal intended velocity. The general warm-up in all sessions included 5 min of jogging and 5 min of joint mobilization exercises. Then, a specific warm-up was performed before each training exercise; this included 2 sets of 8 down to 6 repetitions (2 min rests), with lower loads at the maximal scheduled load in each session. In addition to the training program described above, the participants also performed abdominal and lower-back strengthening exercises.

#### Swimming Training

Both experimental groups performed the same swimming training classified under different types. The training classification system devised for swimmers at the Australian Institute of Sport was used: (a) aerobic maintenance (A2; intensity: 75–80% VO2MAX; heart rate: ~150 bpm); (b) aerobic development (A3; intensity: 80–85% VO2MAX; heart rate: ~170 bpm); (c) maximal aerobic (VO2MAX; intensity: 95–100% VO2MAX; heart rate: ~180–190 bpm); and (d) sprint (A2; intensity: >100% VO2MAX; heart rate: n/a). Additionally, other types of training were also carried out: (e) lower limb training (propulsion was performed only using leg movements); (f) technical training (exercises of swimming styles); (g) test (short-distance sets at competition-level intensities and distances); and (h) hypoxia training (short-distance sets without breathing).

### 2.5. Statistical Analysis

Standard statistical methods were used for the calculation of mean values and standard deviations. Normality of data distribution was examined using the Shapiro–Wilk test, and homogeneity of variance was verified using Levene’s test. Correlations were reported using Pearson product-moment correlation coefficients (r). A one-way random-effects model (model 2,1) ICC with absolute agreement was used to determine the relative reliability. Absolute reliability was reported using the CV. The training-related effects were assessed using a 2 × 2 mixed factorial ANOVA with group (low-load vs. moderate-load) as the between-subject factor and time (pre-training vs. post-training) as the within-subject factor. The group × time interaction was used to determine whether pre-to-post changes differed between groups. When appropriate, Bonferroni-adjusted pairwise comparisons were used to examine within-group pre-to-post changes and between-group differences at each time point. Percentage changes (Δ%) were calculated for descriptive purposes and were not used as an inferential statistical test. Intra- and between-group effect sizes (ES) were calculated using Hedge’s g [10], with threshold values of 0.20, 0.60, 1.20 and 2.00 for small, moderate, large, and very large standardized effects, respectively [11]. Intra-group effect size was calculated as follows: (mean Post − mean Pre)/combined SD, whereas the between-group ES was calculated as follows: (mean G40–50% − mean G55–65%)/combined SD. Statistical analyses were performed using SPSS software version 24.0 (SPSS Inc., Chicago, IL, USA), with *p* < 0.05 indicating statistical significance.

## 3. Results

### 3.1. Jump, Strenght and Swimming Performance

**Figure 1:**
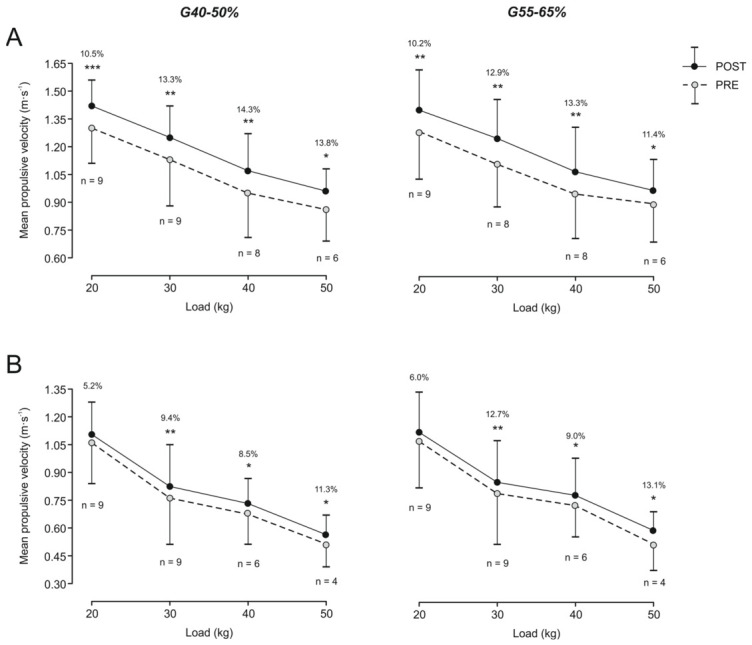
Load–velocity curve in (**A**) the full squat and (**B**) the bench press exercises obtained before and after a 8-week training period. Values are mean ± SD. Significant differences are indicated as follows: * *p* < 0.05, ** *p* < 0.01; *** *p* < 0.001. Note: The sample size in each load was decreased because the participants did not need to progress to that resistance during the initial isoinertial loading test in both the full squat and bench press exercises.

**Figure 2:**
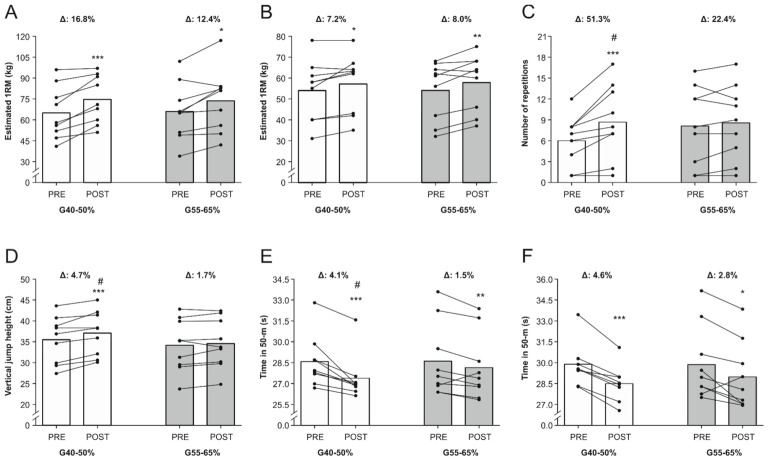
Individual and average Pre and Post values in selected neuromuscular performance variables: estimated 1RM in the full squat exercise (**A**), estimated 1RM in the bench press exercise (**B**), number of repetitions in the pull-up exercise (**C**), jump height in the CMJ test (**D**) time in 50 m freestyle from the starting block (**E**) and time in 50 m freestyle from inside the pool (**F**) for the G40–50% and G55–65% groups. Significant differences are indicated as follows: * *p* < 0.05, ** *p* < 0.01; *** *p* < 0.001 within group and # *p* < 0.05 for “test x group” interaction.

The data for all variables analyzed was homogeneous and normally distributed (*p* < 0.05). At baseline, there were no significant differences in any variable between groups. In both groups, 100% of participants were compliant with the RT program. The average values from pre- to post-training and intra- and between-group ES for all variables analyzed are reported in Table 3. Significant “test x groups” were observed for CMJ, MNR_PU and T50_SB in favor of the G40–50%. There were no significant differences between groups at Post in any variable analyzed. Pre−Post comparisons revealed significant (*p* < 0.05–0.001) changes for G40–50% in all variables analyzed (Table 3), except for MPV20 in the BP exercise (Figure 1). The G55–65% showed significant differences in 1RM_SQ (*p* < 0.05), 1RM_BP (*p* < 0.05), T50_SB (*p* < 0.05), T50_IP (*p* < 0.001), and MPV attained with different absolute loads in the SQ and BP exercises. In addition, G40–50% presented greater intra-group ES (Table 3) and percentage of changes (Figure 1 and Figure 2) compared with G55–65% in all variables analyzed, except in the variables obtained in the BP exercise.

### 3.2. Correlations

When data were pooled, a significant negative correlation (*p* < 0.05–0.01) was observed between individual relative changes in the 50 m swimming tests and the relative changes in CMJ (r = −0.62 and r = −0.63 for T50_SB and T50_IP, respectively) and estimated 1RM in the SQ exercise (r = −0.54 and r = −0.61 for T50_SB and T50_IP, respectively). In addition, a moderate but not significant correlation coefficient was observed between the relative changes in the T50_SB and the relative changes in the MNR in the PU exercise. The relative changes in the estimated 1RM in the BP and PU exercises did not show a significant relationship with the relative changes in the 50 m swimming tests (Table 4).

### 3.3. Quantification of the Swimming Training

During the 8-week training period, all participants performed 48 swimming training sessions (6 sessions per week). Participants swam a total of 445.5 km during the experimental period, which averages out to 55.7 ± 6.9 km per week and 9.3 ± 3.5 km per training session. Specifically, aerobic maintenance intensity (A2) training was done at an average of 35.6 ± 5.6 km per week, whereas aerobic development intensity (A3) was done at an average of 4.8 ± 1.8 km per week. Meanwhile, swimmers spent 1.1 ± 1.3 km and 1.3 ± 0.3 km per week training VO2MAX and maximal speed, respectively. Lower limb exercises and technical training were done at an average of 6.5 ± 1.6 km and 6.2 ± 3.2 km per week, respectively. During the last two weeks of training, the swimmers performed short-distance hypoxia sets (0.37 ± 0.07 km per week) and competition sets (1.6 km per week). The results related to the training load per week during swimming training are presented in Table 5.

## 4. Discussion

The present study examined the effects of two resistance training programs differing exclusively in relative load used in the SQ and BP exercise on neuromuscular and sprint swimming performance in elite junior swimmers. The main findings indicate that both low- and moderate-load training protocols elicited meaningful improvements in strength, jumping ability, and 50 m freestyle performance. Specifically, both groups improved squat and bench press strength and 50 m freestyle performance, whereas improvements in countermovement jump performance, pull-up strength, and maximal number of pull-up repetitions were observed only in the low-load group. In addition, significant group × time interactions were found for countermovement jump performance, maximal number of pull-up repetitions, and 50 m freestyle performance from the starting block, indicating more favorable pre-to-post changes in the low-load group. Taken together, these findings suggest that the two programs appeared to induce different adaptation profiles.

A key strength of the present design is that exercise selection, training volume, execution velocity, and in-water training load were identical between groups, with relative load being the sole manipulated variable. This high level of experimental control strengthens the interpretation that the observed adaptations were primarily influenced by differences in relative load rather than by confounding training factors [16]. From a practical standpoint, these findings suggest that reducing relative load does not compromise neuromuscular or swimming performance adaptations when training variables are appropriately managed.

The more favorable pre-to-post changes observed in countermovement jump performance, maximal number of pull-up repetitions, and 50 m freestyle performance from the starting block in the low-load group may be partly explained by differences in movement velocity and neuromuscular fatigue associated with each intensity range. Lower relative loads allow higher actual concentric velocities, which may enhance neural adaptations related to motor unit recruitment and rate of force development [24,25]. Conversely, moderate-load training, despite being performed with maximal intended velocity, inevitably results in lower movement velocities and greater mechanical and metabolic stress [18].

Lower neuromuscular fatigue associated with low-load training may also facilitate better recovery between sessions, which is particularly relevant in swimming due to the high frequency and volume of in-water training [3,21]. Previous research has shown that RT protocols inducing high levels of fatigue could impair subsequent training quality for up to 24–48 h, potentially interfering with technical and metabolic swimming adaptations [18]. From this perspective, achieving comparable performance improvements with lower relative loads may reflect a more efficient training stimulus, especially in contexts where cumulative fatigue must be carefully managed. Improvements in lower-limb strength and jumping performance are consistent with the established importance of high-speed lower-body actions for swimming starts and turns [9,10]. The observed associations between changes in countermovement jump height, squat strength, and 50 m freestyle performance further support the contribution of lower-limb neuromuscular adaptations to sprint swimming outcomes [8,33].

Regarding upper-limb performance, both groups improved BP and PU strength, although larger relative gains in PU performance were observed in the lower-load group. This finding likely reflects a greater window for neural adaptation in an exercise with lower initial familiarity rather than a load-specific effect per se [23,29]. The BP exercise was included not as a direct predictor of swimming performance, but as a standardized upper-limb resistance exercise allowing controlled manipulation of relative load and assessment of neuromuscular adaptations.

Despite improvements in upper-limb strength, the absence of strong associations between changes in BP or PU strength and swimming performance is consistent with previous studies [8,34]. This suggests that the transfer of dry-land upper-limb strength gains to swimming performance may require longer intervention periods or additional technical adaptations, particularly in young swimmers [35,36].

Overall, the present findings indicate that low-load, high-velocity RT may be considered a practical alternative to moderate-load RT in elite junior swimmers, particularly for selected outcomes in which more favorable pre-to-post changes were observed. However, this interpretation should be considered cautiously and should not be taken as evidence of general superiority of the low-load condition. The observation that selected adaptations were achieved with relatively lower loads is consistent with contemporary perspectives on training efficiency and the management of resistance training dose in athletes exposed to high concurrent training demands [27]. Although Bonferroni-adjusted pairwise comparisons were used when appropriate, the number of performance outcomes and secondary analyses included in the present study may still increase the risk of type I error. Therefore, some secondary findings, particularly the correlation analyses, should be interpreted as exploratory.

From an applied perspective, coaches working with elite junior swimmers may consider resistance training programs using low relative loads performed with maximal intended concentric velocity as one possible option within dry-land training programs, particularly when the objective is to emphasize rapid force production and high movement velocity. In the present study, training with ~40–50% 1RM was associated with more favorable pre-to-post changes in countermovement jump performance, maximal number of pull-up repetitions, and 50 m freestyle performance from the starting block. These findings suggest that relatively low loads can be used without compromising training effectiveness and may be useful when coaches aim to develop selected neuromuscular qualities while maintaining compatibility with high-volume swimming practice. From a long-term athlete development perspective, prioritizing training efficiency and minimum effective dose strategies may help optimize performance while minimizing potential excessive fatigue in young elite swimmers.

## 5. Limitations

Several limitations should be considered when interpreting the findings. First, the eight-week intervention may have been sufficient to induce neuromuscular adaptations, but it may have been too short to allow the full transfer of dry-land strength gains to swimming-specific technical performance, particularly in upper-limb actions [35,36]. Second, although all concentric actions were performed with maximal intended velocity, repetition-by-repetition movement velocity was not directly monitored during training. This limits the ability to quantify the exact mechanical stimulus, velocity loss, and fatigue accumulation associated with each loading condition [26]. Future studies incorporating velocity-based RT may provide a more accurate description of training load and fatigue accumulation.

Third, the relatively small sample size may have limited the statistical power to detect small-to-moderate between-group effects and increased the risk of type II error. Therefore, non-significant between-group findings should not be interpreted as definitive evidence of equivalence between the two resistance training approaches. Nevertheless, this limitation should be considered alongside the homogeneous elite sample and the strict control of training, nutritional, and recovery conditions, which may have reduced inter-individual variability and strengthened internal validity [5,37]. In addition, menstrual cycle phase was not formally recorded or controlled in the female swimmers; however, female participants were equally distributed between groups and represented a small proportion of the total sample, which likely reduced the potential impact of this uncontrolled factor.

Finally, the findings are specific to elite junior swimmers and should not be directly extrapolated to senior, recreational, or less-trained populations. Differences in training history, neuromuscular maturity, and adaptive potential may lead to different responses to low- and moderate-load resistance training [28,29]. The absence of direct assessments of start and turn performance or detailed stroke biomechanics also limits the interpretation of the mechanisms underlying the improvements in sprint swimming performance [4,32]. In addition, sprint swimming performance was assessed using manual rather than electronic timing, which may have reduced sensitivity to small performance changes in highly trained swimmers.

## 6. Conclusions

Both low- and moderate-load resistance training programs performed with maximal intended velocity elicited meaningful improvements in neuromuscular performance and 50 m freestyle swimming performance in elite junior swimmers. Importantly, the use of relatively low loads (40–50% 1RM) did not compromise training effectiveness. Moreover, low-load resistance training resulted in more favorable pre-to-post improvements in countermovement jump performance, maximal number of pull-up repetitions, and 50 m freestyle performance from the starting block. These findings suggest that low-load, high-velocity resistance training using 40–50% 1RM may represent a practical alternative to moderate-load resistance training for young elite swimmers exposed to high concurrent swimming training demands. From a developmental perspective, this approach may be useful when the objective is to promote selected neuromuscular and sprint-performance adaptations while managing the overall demands of concurrent dry-land and in-water training. However, given the small and highly specific sample, these findings should be interpreted with caution and require confirmation in larger cohorts of competitive swimmers.

## Figures and Tables

**Table 1:** Participant characteristics.

	Age (Years)	BM (kg)	Height (m)	FM (%)
**G40–50%**	15.4 ± 0.7	66.8 ± 8.2	1.72 ± 0.07	11.1 ± 5.1
**G55–65%**	15.7 ± 1.0	65.5 ± 8.3	1.71 ± 0.06	10.9 ± 4.3

G40–50%: Group trained at 40–50% 1-repetition maximum (*n* = 9); G55–65%: Group trained at 55–65% 1-repetition-maximum (*n* = 9); BW: Body mass; FM: Fat mass.

**Table 2:** Characteristics of the strength training program.

	Wk 1		Wk 2		Wk 3		Wk 4		Wk 5		Wk 6		Wk 7		Wk 8	
Exercise	S1	S2	S3	S4	S5	S6	S7	S8	S9	S10	S11	S12	S13	S14	S15	S16
SQ (S × R)	3 × 6	3 × 6	3 × 8	3 × 8	3 × 6	3 × 6	4 × 6	3 × 8	3 × 8	3 × 8	3 × 4	4 × 4	3 × 5	3 × 5	4 × 5	4 × 5
CMJ (S × R)	3 × 5	3 × 4	3 × 5	3 × 4	4 × 5	4 × 4	4 × 5	4 × 4	4 × 5	4 × 4	5 × 5	5 × 4	5 × 5	5 × 4	5 × 5	5 × 4
BP (S × R)	3 × 6	3 × 6	3 × 8	3 × 8	3 × 6	3 × 6	4 × 6	3 × 8	3 × 8	3 × 8	3 × 4	4 × 4	3 × 5	3 × 5	4 × 5	4 × 5
PU (S × %MR)	3 × 50%	3 × 50%	4 × 50%	4 × 50%	3 × 60%	3 × 60%	4 × 60%	4 × 60%	4 × 60%	4 × 60%	3 × 75%	3 × 75%	4 × 75%	4 × 75%	4 × 75%	4 × 75%

Wk: Week; S: Session; SQ: Full squat; CMJ: Countermovement jump; BP: Bench press; PU; Pull-ups; S × R: Sets × repetitions; %MR: Percentage of maximum number of repetitions completed during the pull-ups test.

**Table 3:** Pre- and post-training changes in strength and performance variables for each group.

	G40–50%			G55–65%			Changes Between G40–50% vs. G55–65%	
Variables	PRE	POST	ES_IN_ (95% CI)	PRE	POST	ES_IN_ (95% CI)	∆ (%)	ES_BE_ (95% CI)
CMJ (cm) #	35.5 ± 5.6	37.1 ± 5.3 ***	0.29 (0.10 to 0.48)	34.2 ± 6.3	34.6 ± 6.0	0.08 (−0.03 to 0.19)	3.1	0.19 (−0.74 to 1.12)
1RM_SQ (kg)	65.0 ± 18.9	74.7 ±17.3 ***	0.53 (0.20 to 0.87)	66.0 ± 20.6	73.7 ± 22.7 **	0.35 (0.08 to 0.63)	3.1	0.10 (−0.82 to 1.02)
1RM_BP (kg)	54.0 ± 14.6	57.4 ± 14.1 *	0.24 (0.03 to 0.45)	54.1 ± 14.0	57.9 ± 13.5 **	0.27 (0.06 to 0.49)	−0.6	−0.02 (−0.95 to 0.90)
1RM_PU (kg)	10.8 ± 8.9	13.4 ± 9.5 *	0.28 (−0.05 to 0.62)	11.3 ± 9.0	13.1 ± 10.5	0.18 (−0.04 to 0.41)	10.5	0.08 (−0.84 to 1.01)
MNR_PU#	6.0 ± 3.8	8.7 ± 5.5 ***	0.56 (0.23 to 0.90)	8.1 ± 5.8	8.6 ± 5.6	0.18 (−0.08 to 0.23)	37.0	0.42 (−0.51 to 1.36)
T50_SB (s) #	28.57 ± 1.85	27.39 ± 1.62 ***	0.68 (0.26 to 1.10)	28.60 ± 2.64	28.15 ± 2.38 *	0.18 (0.02 to 0.35)	2.6	0.33 (−0.60 to 1.27)
T50_IP (s)	29.89 ± 1.52	28.49 ± 1.26 ***	1.00 (0.47 to 1.53)	29.86 ± 2.70	28.98 ± 2.44 **	0.34 (0.04 to 0.64)	1.8	0.25 (−0.68 to 1.18)

Data are mean ± SD; Pre: initial evaluations; Post: final evaluations; CI: confidence interval; CMJ: countermovement jump height; G40–50%: group trained at 40–50% 1RM (*n* = 9); G55–65%: group trained at 55–65% 1RM (*n* = 9); ES_IN_: intra-group effect size; ES_BE_: between-group effect size; MNR: maximal number of repetitions completed; ∆: percentage of change; 1RM: one-repetition maximum; SQ: full-squat exercise; BP: bench press exercise; PU: pull-up exercise; T50_SB: 50 m swimming time from the starting block; T50_IP: 50 m swimming time from inside the pool. Statistically significant “time × group” interaction; # *p* < 0.05. Intra-group significant differences from pre-to post-training: * *p* < 0.05, ** *p* < 0.01, *** *p* < 0.001. Note: The positive ES indicates a positive effect, whereas the negative ES indicates a negative effect.

**Table 4:** Relationship between the individual relative changes in 50 m freestyle time tests and the individual relative changes in the different performance variables.

Variables	CMJ	1RM_SQ	1RM_BP	1RM_PU	MNR_PU
T50_SB	−0.62 **	−0.54 *	−0.31	−0.35	−0.52
T50_IP	−0.63 **	−0.61 **	−0.40	−0.43	−0.43

1RM: one-repetition maximum; CMJ: countermovement jump height; SQ: full-squat exercise; BP: bench press exercise; PU: pull-up exercise; MNR: maximum number of repetitions completed; T50_SB: 50 m swimming time from the starting block; T50_IP: 50 m swimming time from inside the pool. Statistically significant relationships: * *p* < 0.05, ** *p* < 0.01.

**Table 5:** Total distance per week and during the whole training period (m) for each type of training.

Type of Training	Wk-1	Wk-2	Wk-3	Wk-4	Wk-5	Wk-6	Wk-7	Wk-8	Total
A2 (m)	29,800	33,400	44,320	44,030	31,900	35,650	33,380	32,250	284,730
(%)	(55.2)	(58.4)	(66.9)	(67.3)	(67.1)	(66.5)	(65.1)	(64.2)	(63.9)
A3 (m)	5600	6600	6700	5550	2000	5300	4400	2200	38,350
(%)	(10.4)	(11.5)	(10.1)	(8.5)	(4.2)	(9.9)	(8.6)	(4.4)	(8.6)
VO2_MAX_ (m)	0	0	0	0	1200	2050	3600	1600	8450
(%)	0	0	0	0	(2.5)	(3.8)	(7.0)	(3.2)	(1.9)
Speed training (m)	840	840	1700	1400	1650	1250	1585	1120	10,385
(%)	(1.6)	(1.5)	(2.6)	(2.1)	(3.5)	(2.3)	(3.1)	(2.2)	(2.3)
Lower limb (m)	6200	6200	7000	9900	4900	6800	4550	6200	51,750
(%)	(11.5)	(10.8)	(10.6)	(15.1)	(10.3)	(12.7)	(8.9)	(12.3)	(11.6)
Technical (m)	11,560	10,160	6500	4500	5900	2600	3415	4880	49,515
(%)	(21.4)	(17.8)	(9.9)	(6.9)	(12.4)	(4.9)	(6.7)	(9.7)	(11.1)
Hypoxia (m)	0	0	0	0	0	0	320	420	740
(%)	0	0	0	0	0	0	(0.6)	(0.8)	(0.2)
Test (m)	0	0	0	0	0	0	0	1600	1600
(%)	0	0	0	0	0	0	0	(3.2)	(0.4)
**Total**	**54,000**	**57,200**	**66,220**	**65,380**	**47,550**	**53,650**	**51,250**	**50,270**	**445,520**

Wk: Week; A2: Training at aerobic maintenance intensity; A3: Training at aerobic development intensity; VO2MAX: Maximum oxygen consumption; Test: Training at intensity of competition; Technical: Exercises of swimming styles; Test: Short-distance sets at intensity of competition; Hypoxia: Short-distance sets without breathing.

## Data Availability

The raw data supporting the conclusions of this article will be made available by the authors, without undue reservation.

## Abbreviations

The following abbreviations are used in this manuscript:

RT	Resistance Training
1RM	1 Repetition Maximun
ES	Effect Size
BW	Body Weigth
FM	Fat Mass
CMJ	Countermovement Jump
BP	Bench Press
SQ	Full Squat
MPV	Concentric Mean Propulsive Velocity
PU	Pull Exercise
